# The gender difference and mortality-to-incidence ratio relate to health care disparities in bladder cancer: National estimates from 33 countries

**DOI:** 10.1038/s41598-017-04083-z

**Published:** 2017-06-28

**Authors:** Shao-Chuan Wang, Wen-Wei Sung, Yu-Lin Kao, Tzuo-Yi Hsieh, Wen-Jung Chen, Sung-Lang Chen, Horng-Rong Chang

**Affiliations:** 1 Department of Urology, Chung Shan Medical University Hospital, Taichung, Taiwan; 2 School of Medicine, Chung Shan Medical University, Taichung, Taiwan; 3 Institute of Medicine, Chung Shan Medical University, Taichung, Taiwan; 4 Department of Medical Education, Chung Shan Medical University Hospital, Taichung, Taiwan; 5 Department of Medical Technology, Jen-Teh Junior College of Medicine, Nursing and Management, Miaoli, Taiwan; 6 Division of Nephrology, Department of Internal Medicine, Chung Shan Medical University Hospital, Taichung, Taiwan

## Abstract

The variation in the mortality-to-incidence ratio (MIR) between countries and genders reflects the complex etiology and intervention of bladder cancer. In this study, we investigated the MIR variation between genders and health care disparities among countries. Cancer incidence and mortality were obtained from the GLOBOCAN 2012 database. The ranking and the total expenditure on health of countries were obtained from the World Health Organization. Linear regression was used to estimate the significance between variables. We estimated the role of MIRs from 33 countries. Bladder cancer incidence and mortality rates were higher in more developed regions, Europe, and the Americas. The MIRs were higher in less developed regions. Analysis according to country revealed Germany to have the lowest MIR. High relative MIRs (female MIR/male MIR) for bladder cancer were noted in many developed countries. A correlation between MIR and health care disparities among countries was indicated by a significant association between the World Health Organization ranking and total expenditure on health/GDP with the MIR and relative MIR. Low bladder cancer MIR is prone to be more prevalent in countries with good health care system.

## Introduction

Bladder cancer shows a high incidence and mortality that differs with gender, race, and ethnicity^1, 2^. Moreover, the incidence and mortality rates vary across countries due to the differences in risk factors, detection, diagnostic practices, and treatments^2–5^. A recent global study showed that the incidence and mortality rates had decreased in most Western countries but had increased in some eastern European and developing countries^2, 6^. This evidence confirms the need for continued surveillance and analysis of the patterns and trends in bladder cancer incidence, mortality, and survival among countries.

The surveillance of the epidemiology of cancers often involves analysis of their incidence and mortality. The mortality-to-incidence ratio (MIR) is a novel measure that can evaluate the cancer mortality in relation to incidence; it serves as a proxy for 1-survival^7, 8^. Asadzadeh Vostakolaei F. and his colleagues demonstrated that the ratio appears to be a fairly accurate simple predictor of 5-year survival rates^8^. It was recently used to identify whether a country has a higher mortality than expected based on its incidence^7–9^. The MIR has also proved useful for identifying disparities in cancer screening and treatment for colorectal cancer^9^. Its potential as an indicator in the long-term success of cancer surveillance programs has been impressive^9^. Previous study on sex differences in bladder cancer using the GLOBOCAN 2008 database demonstrated an increased mortality in women compared to men as an apparently common phenomenon^10^. Of the 49 countries analyzed, 53% had relative MIR (relative MIR: female MIR/male MIR) values greater than one^10^. By contrast, only 4% of the countries had relative MIR values less than one.

The release of updated data in the GLOBOCAN 2012 database prompted us to follow up on the trends in bladder cancer, focusing especially on this gender difference. In addition, no report has yet investigated continental differences in the relative MIR or provided a comparison between developed and developing regions. In this study, we follow up the trends of MIR between countries and regions with the database of GLOBOCAN 2012. Additionally, we analyzed the relative MIR according to the region development, human development, and continents. We also included the World Health Organization ranking (WHO ranking) and total expenditure on health/GDP (e/GDP) to investigate health care disparities between countries, in order to understand the contribution of the MIR to the health care system. We hypothesize that the MIR of bladder cancer could reflect disparities in health care among countries and that the gender difference observed for the MIR in bladder cancer remains an important issue today.

## Materials and Methods

The data acquisition was described previously^11^. Data were obtained from the GLOBOCAN 2012 database, maintained by the International Agency for Research on Cancer. The GLOBOCAN 2012 database is public access and provides contemporary estimates of the incidence of, mortality and prevalence from major types of cancer for 184 countries of the world. The categories of countries’ developing were obtained from this database. Detailed summarized data from GLOBOCAN 2012 can be found in the article by Torre LA and his colleagues^12^. The included criteria for the selected countries of this investigation were the availability/quality of cancer incidence and mortality information separately for men and women. We excluded countries due to lack of WHO ranking data (22 countries); low availability/quality level of data (ranking E to G for incidence or ranking 4 to 6 for mortality, 105 countries); incidence number less than 1,000. Overall, 33 countries met these criteria and were included in our analysis. Crude rates were defined as the rates per 100,000. The WHO ranking was obtained from World Health Organization’s Ranking of the World’s Health Systems, maintained by the World Health Organization. The e/GDP and life expectancy for 2012 were obtained from the World Health Statistics 2015, which is an annual compilation of health-related data for its 194 member states.

The method of statistical analyses was described previously^11^. The MIR is defined as the percentage given by the ratio of the crude rate of mortalities and the crude rate of incidences. The relative MIR is defined as the ratio of the female MIR to the male MIR^10^. The associations between the MIR and other factors among countries were estimated using simple linear regression via SPSS statistical software (version 15.0) (SPSS, Inc., Chicago, IL). A value of p < 0.05 was considered statistically significant. Scatter plots were generated using Microsoft Excel 2010.

## Results

### The high crude rate of bladder cancer incidence and mortality in more developed regions, Europe, and the Americas

We sought a better understanding of the global trends in bladder cancer by analyzing the incidence and mortality according to different regions. The results are summarized in Table 1. The overall crude rates of incidence and mortality were 6.1 and 2.3, respectively. Comparison according to the development level of the different regions revealed a higher crude rate of incidence and mortality in the more developed regions than in the less developed regions (incidence: 20.4 vs 3.0, and mortality: 6.4 vs 1.5, respectively). The trends were the same for both genders. We further analyzed the crude rates according to WHO regions and continent and found the WHO Europe region to have the highest incidence and mortality (incidence/mortality: 18.5/6.5). Among the different continents, North America had the highest incidence crude rate (21.9), but the highest mortality crude rate was found for Europe (7.1). These results showed that bladder cancer has a higher incidence and mortality crude rates in regions with high development, particularly North America and Europe.

**Table 1:** Summary of bladder cancer crude rate of incidence, mortality, and mortality-to-incidence ratio of 184 countries according to regions.

Region	Incidence, number			Mortality, number			Incidence, crude rate^1^			Mortality, crude rate^1^			Mortality-to-incidence ratio			Relative MIR
	Total	Female	Male	Total	Female	Male	Total	Female	Male	Total	Female	Male	Total	Female	Male	
World	429,793	99,413	330,380	165,084	42,033	123,051	6.1	2.8	9.3	2.3	1.2	3.5	0.38	0.43	0.38	1.14
Development																
More developed regions	253,843	57,766	196,077	79,938	21,024	58,914	20.4	9.0	32.4	6.4	3.3	9.7	0.31	0.37	0.30	1.22
Less developed regions	175,950	41,647	134,303	85,146	21,009	64,137	3.0	1.5	4.5	1.5	0.7	2.2	0.50	0.47	0.49	0.95
WHO region categories																
Africa region	11,853	4,189	7,664	6,993	2,619	4,374	1.3	1.0	1.7	0.8	0.6	1.0	0.62	0.60	0.59	1.02
Americas region	101,593	25,894	75,699	28,739	8,376	20,363	10.7	5.4	16.1	3.0	1.7	4.3	0.28	0.31	0.27	1.18
East Mediterranean region	27,690	5,789	21,901	13,822	2,932	10,890	4.4	1.9	6.9	2.2	1.0	3.4	0.50	0.53	0.49	1.07
Europe region	166,583	35,143	131,440	58,758	13,812	44,946	18.5	7.6	30.1	6.5	3.0	10.3	0.35	0.39	0.34	1.15
South-East Asia region	30,708	6,302	24,406	17,139	3,562	13,577	1.7	0.7	2.6	0.9	0.4	1.4	0.53	0.57	0.54	1.06
Western Pacific region	91,294	22,083	69,211	39,606	10,727	28,879	5.0	2.5	7.3	2.1	1.2	3.1	0.42	0.48	0.42	1.13
Continent																
Africa	24,437	6,752	17,685	13,268	3,906	9,362	2.3	1.3	3.3	1.2	0.7	1.7	0.52	0.54	0.52	1.05
Latin America and Caribbean	24,844	7,234	17,610	10,147	3,069	7,078	4.1	2.4	5.9	1.7	1.0	2.4	0.41	0.42	0.41	1.02
Northern America	76,749	18,660	58,089	18,592	5,307	13,285	21.9	10.5	33.5	5.3	3.0	7.7	0.24	0.29	0.23	1.24
Asia	148,568	32,922	115,646	69,294	16,478	52,816	3.5	1.6	5.3	1.6	0.8	2.4	0.46	0.50	0.45	1.10
Europe	151,297	32,932	118,365	52,411	12,889	39,522	20.4	8.6	33.1	7.1	3.4	11.1	0.35	0.40	0.34	1.18
Oceania	3,898	913	2,985	1,372	384	988	10.3	4.8	15.8	3.6	2.0	5.2	0.35	0.42	0.33	1.27

^1^Crude rates were defined as the rates per 100,000.

### The mortality-to-incidence ratio of bladder cancer is high in less developed regions

The MIR demonstrates the related outcome of patients with a specific disease; therefore, we investigated the MIR according to regions. The results are listed in Table 1. The global MIR for bladder cancer was 0.38. Contrary to the findings in crude rate, the less developed regions had higher MIR values when compared to the more developed regions (0.50 and 0.31, respectively). The MIRs were higher in the WHO Africa region, the East Mediterranean region, and the Southeast Asia regions. Among the continents, Africa had the highest MIR compared to other regions (0.52). Therefore, less developed regions, African regions, the East Mediterranean region, and Southeast Asian regions had high MIR values.

A previous study that used the GLOBOCAN 2008 database found a gender difference between MIR in bladder cancer but not in kidney cancer^10^. We investigate the change in gender difference in MIR with the updated database using the relative MIR (MIR in female/MIR in male). The relative MIR for bladder cancer was high in more developed regions (relative MIR: 1.22). Comparison of the WHO region categories and continental distributions revealed that the relative MIRs were high in the WHO American regions (1.18) and North America (1.24). However, no difference was observed for the relative MIR for kidney cancer (data not shown). These results indicated that the MIRs are higher in less developed regions and in Africa. A gender difference was also evident for the MIR for bladder cancer.

### Germany has the lowest mortality-to-incidence ratio in bladder cancer

We further compared the differences in epidemiology among 33 countries. The crude rate of incidence and mortality of bladder cancer are summarized in Table 2. The highest incidence and mortality rates were found for Belgium and Spain, respectively. The case numbers of incidence and mortality are listed in the Supplementary Table 1. As to the MIR and relative MIR according to the countries, United Kingdom has the highest MIR (0.56), while the Germany has the lowest MIR (0.19). The relative MIR values were higher than 1.4 for Germany, Japan, France, Republic of Korea, Austria, Switzerland, and Colombia. No country had a relative MIR value greater than 1.25 for kidney cancer (data not shown).

**Table 2:** Summary of World Health Organization ranking, total expenditure on health, life expectancy, bladder cancer incidence, mortality, and mortality-to-incidence ratio of 33 countries.

Country	Ranking	Total expenditure on health/GDP (%)	Life expectancy	Incidence, crude rate^1^			Mortality, crude rate^1^			Mortality-to-incidence ratio			Relative MIR
				Total	Female	Male	Total	Female	Male	Total	Female	Male	
France	1	11.6	82	17.6	5.8	30.0	7.5	3.4	11.8	0.43	0.59	0.39	1.49
Italy	2	9.2	83	30.0	11.6	49.1	9.4	3.9	15.2	0.31	0.34	0.31	1.09
Spain	7	9.3	83	29.5	9.3	50.1	10.7	3.8	17.7	0.36	0.41	0.35	1.16
Austria	9	11.1	81	25.6	12.4	39.5	5.8	3.7	7.9	0.23	0.30	0.20	1.49
Japan	10	10.3	84	17.4	8.1	27.2	6.0	3.8	8.4	0.34	0.47	0.31	1.52
Norway	11	9.3	82	27.8	14.4	41.1	6.7	4.1	9.3	0.24	0.28	0.23	1.26
Portugal	12	9.9	81	26.9	9.7	45.1	8.0	3.6	12.7	0.30	0.37	0.28	1.32
Netherlands	17	12.7	81	17.9	8.9	27.1	7.5	4.7	10.3	0.42	0.53	0.38	1.39
United Kingdom	18	9.3	81	14.0	7.6	20.5	7.9	5.2	10.6	0.56	0.68	0.52	1.32
Switzerland	20	11.4	83	27.3	13.0	42.2	6.2	3.9	8.6	0.23	0.30	0.20	1.47
Belgium	21	10.9	80	40.3	15.8	65.8	9.2	4.6	13.9	0.23	0.29	0.21	1.38
Colombia	22	6.8	78	2.6	1.4	4.0	1.1	0.7	1.4	0.42	0.50	0.35	1.43
Sweden	23	9.6	82	24.7	12.1	37.5	7.2	4.2	10.3	0.29	0.35	0.27	1.26
Germany	25	11.3	81	34.6	16.2	53.8	6.6	4.5	8.8	0.19	0.28	0.16	1.70
Israel	28	7.4	82	18.4	7.2	29.8	4.0	2.0	6.0	0.22	0.28	0.20	1.38
Canada	30	10.9	82	23.3	12.1	34.7	6.1	3.7	8.6	0.26	0.31	0.25	1.23
Finland	31	9.1	81	20.2	8.1	32.7	5.1	2.6	7.7	0.25	0.32	0.24	1.36
Australia	32	8.9	83	15.2	7.1	23.4	4.7	2.6	6.9	0.31	0.37	0.29	1.24
Denmark	34	11.0	80	31.8	16.7	47.3	10.0	6.5	13.5	0.31	0.39	0.29	1.36
United States of America	37	17.0	79	21.7	10.4	33.4	5.2	2.9	7.6	0.24	0.28	0.23	1.23
Cuba	39	8.6	78	13.3	4.9	21.5	6.3	3.0	9.6	0.47	0.61	0.45	1.37
Thailand	47	4.5	75	3.6	1.7	5.6	1.8	0.9	2.8	0.50	0.53	0.50	1.06
Czech Republic	48	7.5	78	23.3	12.8	34.2	7.0	4.3	9.8	0.30	0.34	0.29	1.17
Poland	50	6.8	77	20.8	9.3	33.1	8.5	3.5	14.0	0.41	0.38	0.42	0.89
Korea, Republic of	58	7.6	82	8.4	3.2	13.6	2.7	1.5	3.9	0.32	0.47	0.29	1.63
Egypt	63	4.9	71	10.6	4.8	16.4	5.2	2.4	8.0	0.49	0.50	0.49	1.03
Belarus	72	5.0	72	11.3	4.1	19.7	4.1	1.4	7.2	0.36	0.34	0.37	0.93
Argentina	75	6.8	76	8.6	4.0	13.4	3.5	1.6	5.5	0.41	0.40	0.41	0.97
Ukraine	79	7.5	71	11.0	3.8	19.5	5.0	1.3	9.4	0.45	0.34	0.48	0.71
Bulgaria	102	7.4	75	22.5	9.9	35.9	7.5	3.0	12.4	0.33	0.30	0.35	0.88
Brazil	125	9.5	75	5.0	2.9	7.2	2.0	1.2	2.9	0.40	0.41	0.40	1.03
Russian Federation	130	6.5	69	9.7	4.0	16.4	4.8	1.9	8.2	0.49	0.48	0.50	0.95
South African Republic	175	8.9	60	3.3	1.8	4.9	1.3	0.8	1.9	0.39	0.44	0.39	1.15

^1^Crude rates were defined as the rates per 100,000.

**Figure 1:**
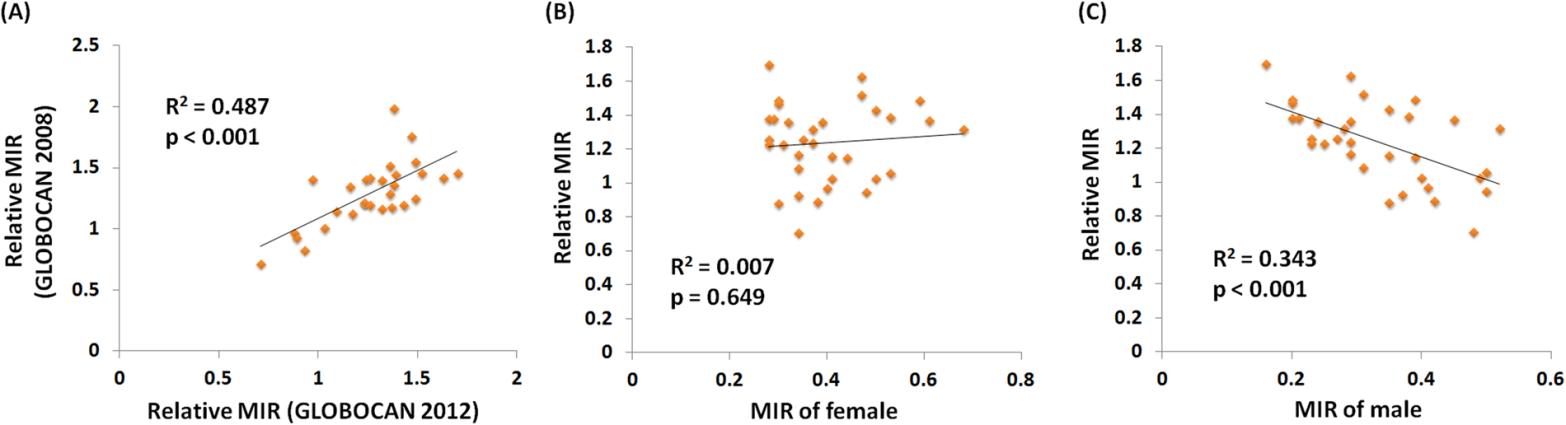
The scatterplots evidenced the relative MIR calculated using the GLOBOCAN 2012 database is positively related with that obtained using the GLOBOCAN 2008 database (n = 29) (**A**). The scatterplots correlate the relative MIR with (**B**) female MIR and (**C**) male MIR.

The gender difference for the MIR in bladder cancer was evident in countries distributed on different continents. A further comparison of the relation between the current relative MIR and previously published relative MIR for bladder cancer revealed that countries with high relative MIR according to the GLOBOCAN 2008 database were prone to have high relative MIR according to the GLOBOCAN 2012 database (R^2^ = 0.487, p < 0.001, Fig. 1A)^10^. Interestingly, the relative MIR values significantly correlated with the male MIR values but not the female MIR values (R^2^ = 0.007, p = 0.649 for female; R^2^ = 0.343, p < 0.001 for male, Fig. 1B and C).

### World Health Organization ranking and total expenditure on health/GDP are significantly associated with the mortality-to-incidence ratios in bladder cancer

**Figure 2:**
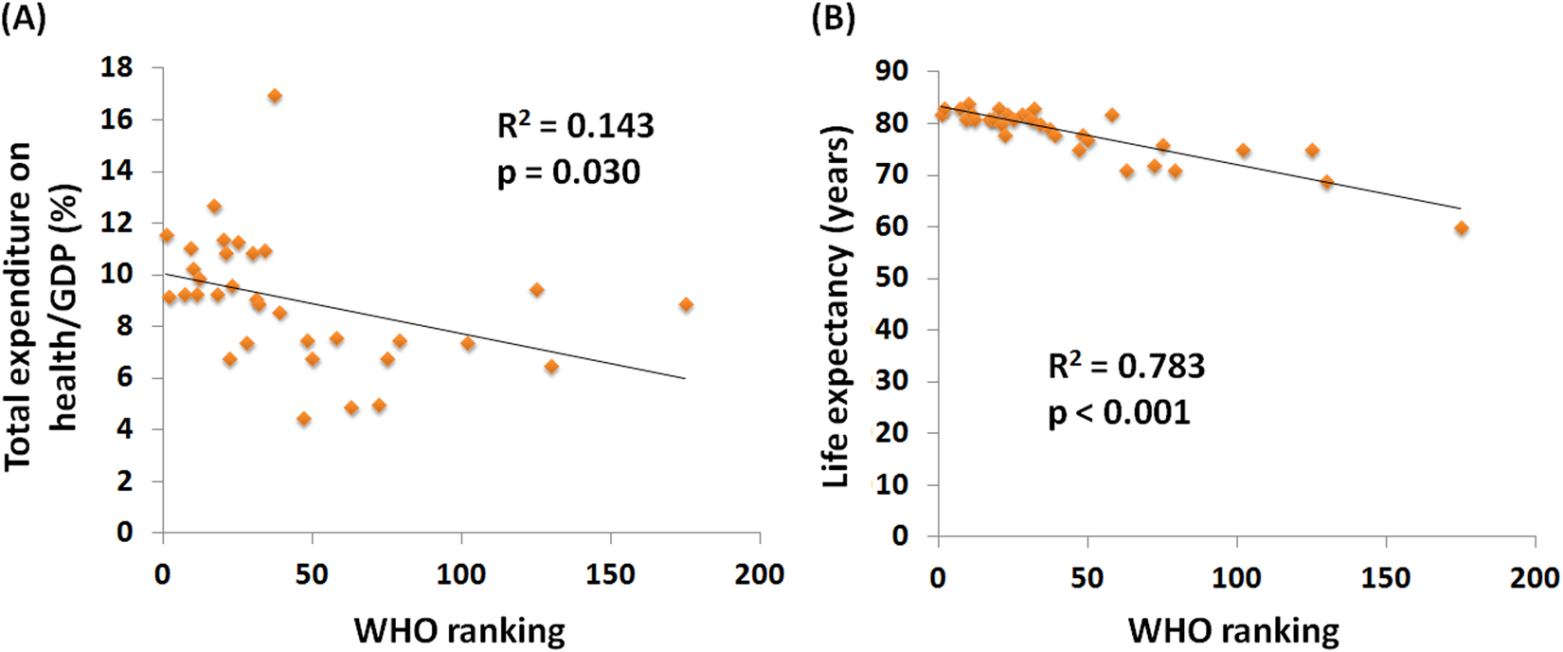
The association between world health organization ranking and (**A**) total expenditure on health/GDP and (**B**) life expectancy among 33 countries.

**Figure 3:**
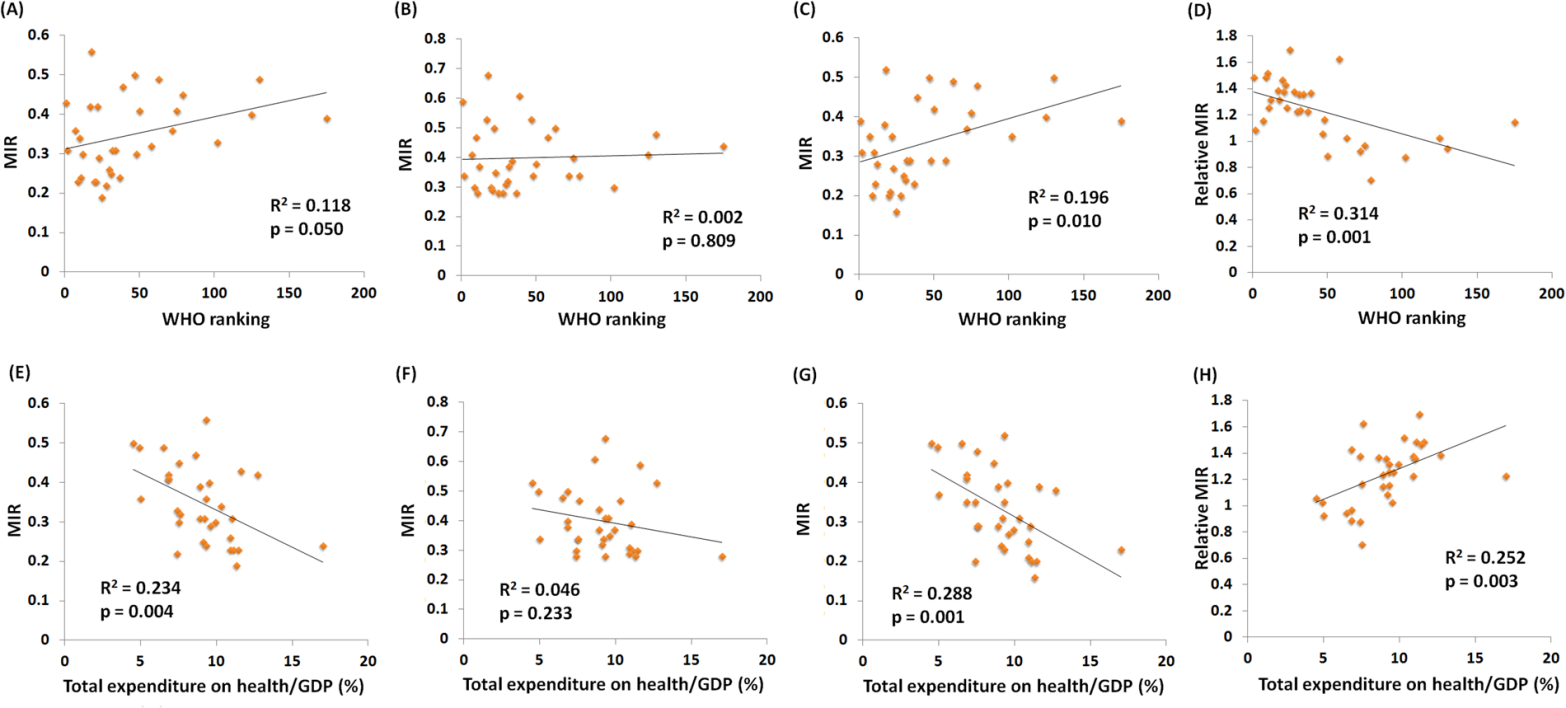
Countries with good world health organization ranking have low MIR in (**A**) both genders, (**B**) female, and (**C**) male, but have high relative MIR (**D**). Countries with high total expenditure on health/GDP have a relatively low MIR in (**E**) both genders, (**F**) female, and (**G**) male, but high relative MIR (**H**).

The MIR is used for the evaluation of health care disparities, so we investigated the correlation between the MIR and WHO ranking and the e/GDP. The information about the WHO ranking, e/GDP, and life expectancy is summarized in Table 2. As expected, the WHO ranking was significantly associated with the e/GDP and the life expectancy (R^2^ = 0.143, p = 0.030, R^2^ = 0.783, p < 0.001, Fig. 2). The WHO ranking and e/GDP were also significantly associated with the crude rate of incidence and mortality (Supplementary Figures 1 and 2). The countries with better ranking or e/GDP showed significant trends for a higher crude rate when compared to other countries. Countries with a high ranking and e/GDP had a low MIR (R^2^ = 0.118, p = 0.050; R^2^ = 0.234, p = 0.004, Fig. 3A and E). However, unlike the significant correlation in male, the correlation between MIR and WHO ranking or e/GDP was not significant in female (Fig. 3B, C, F, and G). The relative MIR, which shows the gender difference in MIR, indicated that a high relative MIR is found in countries with a high ranking or high e/GDP (R^2^ = 0.314, p = 0.001, R^2^ = 0.252, p = 0.003, Fig. 3D and H). The MIRs and relative MIRs are correlated with the WHO ranking and e/GDP, suggesting a role that reflects health disparities.

## Discussion

This is the first study to demonstrate a correlation between the MIR of bladder cancer and the health care disparities among countries. We also followed up the gender differences in the MIR using an updated database. In this study, the WHO ranking and e/GDP were used as indicators of health care disparities. Our findings suggested that the better support of national health care or higher expenditures were correlated with lower MIRs for bladder cancer (Fig. 3). However, countries with good WHO ranking and e/GDP had significantly higher incidences and mortality rates (Supplementary Figures 1 and 2). Our results indicate the importance of health care or the health care system in bladder cancer prognosis, even though detailed information about the differences in treatment interventions between countries was not available to our study. Notably, countries that were more developed, and had good WHO rankings and e/GDP had significantly higher incidences and mortality rates, in agreement with many global investigations^5, 13, 14^. These findings could be associated with occupational and environmental exposures to carcinogens, such as heavy metals, aromatic amines, organic solvents, or indoor pollutants that are known to contribute to the risk of bladder cancer^15–18^. Otherwise, more access to diagnostic procedures such as cystoscopy and transurethral resection in more developed countries might also contribute to this finding. However, in the developing countries, advanced and aggressive bladder cancer patients, who are dying from the disease, would be more prone to be diagnosed and recorded than those with less aggressive disease representing the majority of bladder cancer, which would be the biases the MIR upward.

Among 33 countries, United Kingdom has the highest MIR (0.56, 0.68, and 0.52; total, female, and male, respectively). Compared with previous data analyzed according to the GLOBCAN 2008, the MIR of United Kingdom increased (0.49, 0.62, and 0.44; total, female, and male, respectively)^10^. The reason of high bladder cancer MIR in United Kingdom is unclear. Though, there are 61 countries with higher bladder cancer MIR than the one of United Kingdom among all 184 countries analyzed in GLOBCAN 2012, none of them has better WHO ranking than the ranking of United Kingdom. More detail analysis of the diagnosis, treatment intervention, and death cause of bladder cancer in United Kingdom would be necessary.

The gender difference observed in the incidence and mortality of bladder cancer is an important issue^10, 14^. The updated database revealed a similar phenomenon in our study. Countries with high relative MIR determined from the previous database were also prone to have a high relative MIR according to GLOBOCAN 2012 (Fig. 1A). The underlying etiology of this phenomenon remains unclear, but possible factors could include smoking habits, tumor biology, occupational risk factors, and sex steroid hormones and their receptors^5, 14, 19^. However, we also noticed that countries with a high relative MIR did not have a higher female MIR when compared to other countries. Therefore, we considered that this phenomenon represented a contribution by male patients, but not female. The relative MIRs significantly correlated with the male MIR but not the female MIR, which further supported this idea (Fig. 1A and B).

A recent collaborative review indicated that women suffering from hematuria experienced a significantly greater delay in urologic referral and underwent guideline-concordant imaging less frequently when compared with men^5^. This would reduce the contribution of the health or health care system variance in the MIR. Indeed, the association between the MIR and WHO ranking and e/GDP was not significant in females (Fig. 3). The male patients had more available diagnostic surveys in countries with good health care systems, so the reduction in the MIR contributed to the trend in the relative MIR shown in our study.

The MIR of colorectal and prostate cancer has been confirmed as an indicator of health disparities among countries by the linkage between the MIR and the WHO ranking^9, 11^. In our study, we also included the e/GDP to represent health disparities. To the best of our knowledge, this is the first study to correlate the MIR and e/GDP. As expected, countries with better WHO ranking had higher e/GDP and longer life expectancy. Furthermore, in countries with better health support, the MIRs are significantly lower. Ours findings strengthen the role of the bladder cancer MIR in predicting health care disparities.

One limitation of our study is that not all countries were included in the analysis. The MIR is more available but with more bias in the analysis and collection of database^8^. Therefore, the limitation of using MIR but not the follow up prognosis would be considered and age-standardized rates should be further investigated^8^. Moreover, the different coding of attributed death cause might also affect the result. To reduce this effect, we included countries according to the data quality. The limited information about incidence and mortality meant that the characteristics of clinical stage, treatment intervention, pathology categories, and baseline data were not measured. Furthermore, the use of the WHO ranking and e/GDP to represent the health care or health care system of countries is not sufficiently specific.

In this study, we provided the evidence that the gender difference and low MIR of bladder cancer between countries with good WHO rankings and high e/GDP in their health care systems confirmed the effect of cancer care disparities in the prognosis of bladder cancer. Further investigation with much greater detail and focus data is needed to validate our findings and provide strategy to improved clinical outcome.

## Electronic supplementary material

Supplementary Figures and Table
