# Supplementary material for: The gender difference and mortality-to-incidence ratio relate to health care disparities in bladder cancer: National estimates from 33 countries
Source: Sci Rep. 2017 Jun 28;7:4360. doi: 10.1038/s41598-017-04083-z (PMC5489533; doi:10.1038/s41598-017-04083-z)
Supplement: Supplementary file 1 — Supplementary Figures and Table [file 41598_2017_4083_MOESM1_ESM.doc]

**The gender difference and mortality-to-incidence ratio relate to health care disparities in bladder cancer: National estimates from 33 countries**

Shao-Chuan Wang, Wen-Wei Sung, Yu-Lin Kao, Tzuo-Yi Hsieh, Wen-Jung Chen^1,2,3^, Sung-Lang Chen, Horng-Rong Chang

**Supplementary Figures**


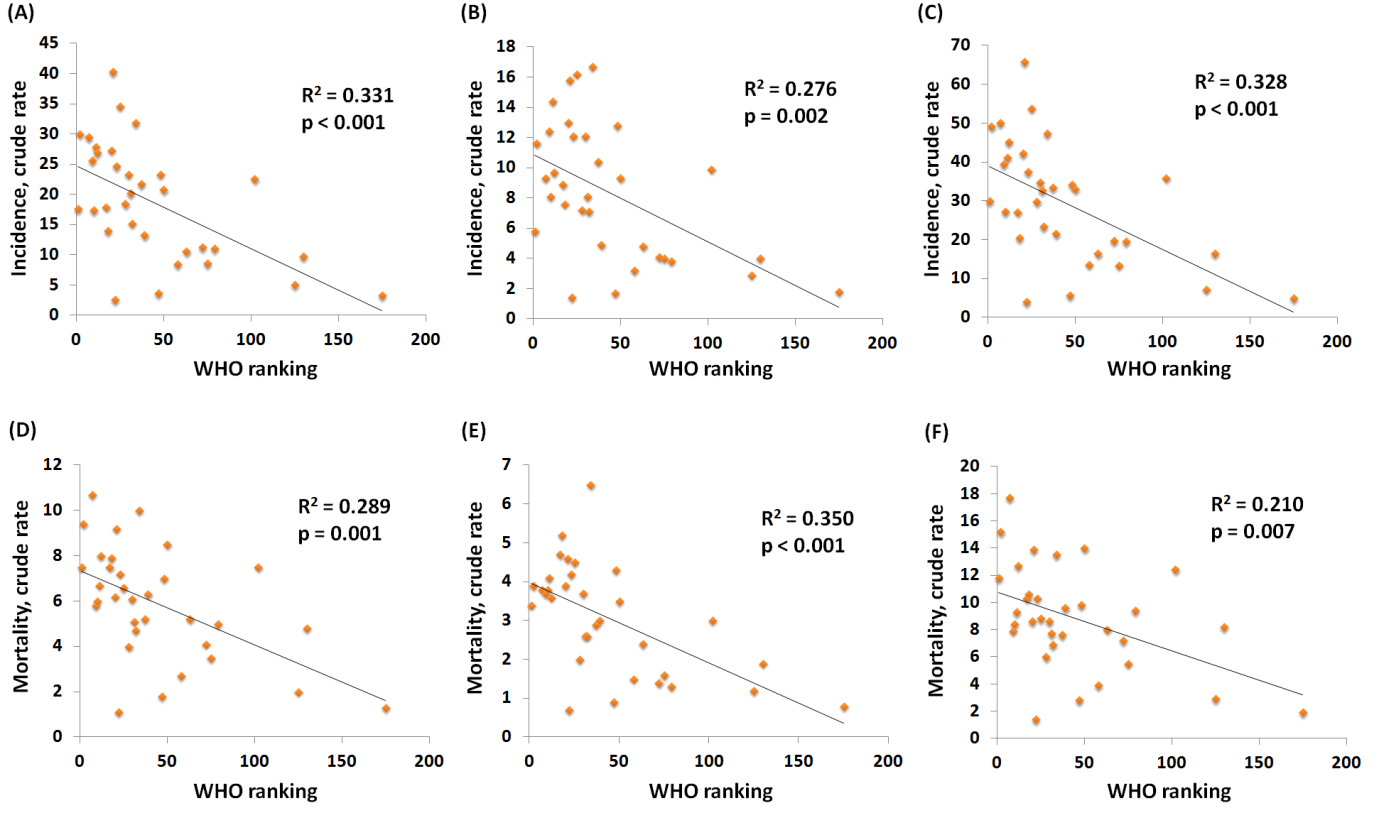
Supplementary Figure 1. Countries with good world health organization ranking have a high crude rate of incidence in (A) both genders, (B) female, and (C) male, as well as a high crude rate of mortality in (D) both genders, (E) female, and (F) male. Crude rates were defined as the rates per 100,000.


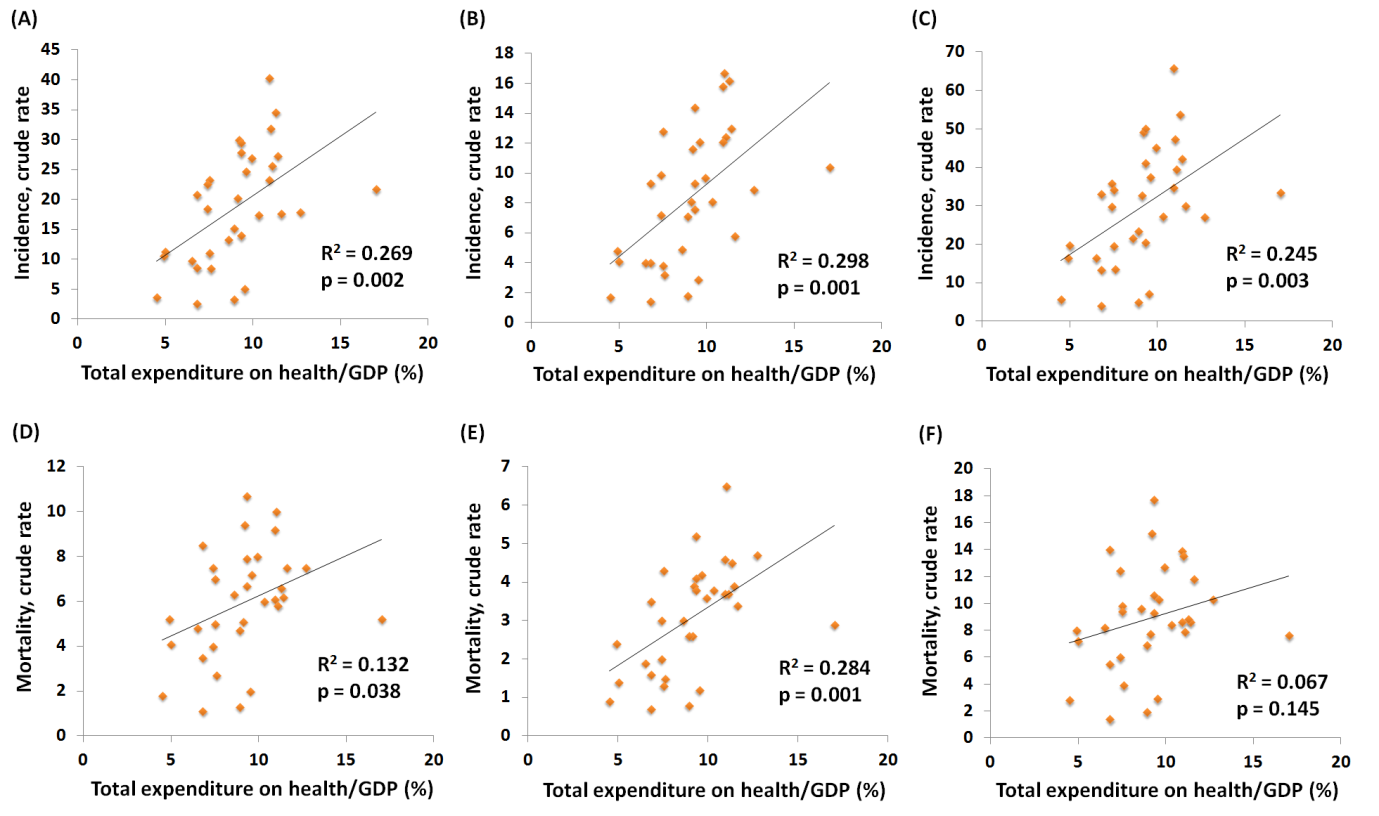
Supplementary Figure 2. Countries with high total expenditure on health/GDP have a high crude rate of incidence in (A) both genders, (B) female, and (C) male, as well as a high crude rate of mortality in (D) both genders, (E) female, and (F) male. Crude rates were defined as the rates per 100,000.

Supplementary Table 1. Summary of bladder cancer number of incidence, and mortality of 33 countries.

|  | Incidence, number | | |  | Mortality, number | | |
| --- | --- | --- | --- | --- | --- | --- | --- |
| Country | Total | Female | Male |  | Total | Female | Male |
| France | 11,175 | 1,892 | 9,283 |  | 4,774 | 1,120 | 3,654 |
| Italy | 18,284 | 3,610 | 14,674 |  | 5,745 | 1,198 | 4,547 |
| Spain | 13,789 | 2,205 | 11,584 |  | 5,007 | 905 | 4,102 |
| Austria | 2,159 | 534 | 1,625 |  | 486 | 159 | 327 |
| Japan | 22,042 | 5,287 | 16,755 |  | 7,630 | 2,462 | 5,168 |
| Norway | 1,378 | 357 | 1,021 |  | 334 | 102 | 232 |
| Portugal | 2,876 | 537 | 2,339 |  | 854 | 196 | 658 |
| Netherlands | 2,999 | 750 | 2,249 |  | 1,253 | 396 | 857 |
| United Kingdom | 8,778 | 2,432 | 6,346 |  | 4,935 | 1,640 | 3,295 |
| Switzerland | 2,115 | 511 | 1,604 |  | 481 | 153 | 328 |
| Belgium | 4,350 | 868 | 3,482 |  | 989 | 255 | 734 |
| Colombia | 1,252 | 327 | 925 |  | 501 | 174 | 327 |
| Sweden | 2,350 | 574 | 1,776 |  | 685 | 199 | 486 |
| Germany | 28,405 | 6,749 | 21,656 |  | 5,404 | 1,861 | 3,543 |
| Israel | 1,414 | 281 | 1,133 |  | 304 | 77 | 227 |
| Canada | 8,086 | 2,114 | 5,972 |  | 2,121 | 638 | 1,483 |
| Finland | 1,093 | 224 | 869 |  | 276 | 71 | 205 |
| Australia | 3,489 | 815 | 2,674 |  | 1,082 | 294 | 788 |
| Denmark | 1,781 | 470 | 1,311 |  | 558 | 183 | 375 |
| United States of America | 68,639 | 16,540 | 52,099 |  | 16,468 | 4,669 | 11,799 |
| Cuba | 1,493 | 276 | 1,217 |  | 713 | 170 | 543 |
| Thailand | 2,537 | 616 | 1,921 |  | 1,288 | 312 | 976 |
| Czech Republic | 2,462 | 688 | 1,774 |  | 741 | 232 | 509 |
| Poland | 7,961 | 1,848 | 6,113 |  | 3,276 | 693 | 2,583 |
| Korea, Republic of | 4,097 | 792 | 3,305 |  | 1,330 | 375 | 955 |
| Egypt | 8,923 | 2,021 | 6,902 |  | 4,381 | 993 | 3,388 |
| Belarus | 1,079 | 207 | 872 |  | 387 | 69 | 318 |
| Argentina | 3,546 | 845 | 2,701 |  | 1,446 | 344 | 1,102 |
| Ukraine | 4,964 | 933 | 4,031 |  | 2,261 | 327 | 1,934 |
| Bulgaria | 1,662 | 380 | 1,282 |  | 558 | 115 | 443 |
| Brazil | 9,924 | 2,877 | 7,047 |  | 4,021 | 1,216 | 2,805 |
| Russian Federation | 13,853 | 3,062 | 10,791 |  | 6,843 | 1,451 | 5,392 |
| South African Republic | 1,695 | 457 | 1,238 |  | 682 | 212 | 470 |
